# Notes and News

**Published:** 1887-06-18

**Authors:** 


### Notes and News.

Collected and Communicated.

His Royal Highness, Prince Albert Victor, has consented to lay the foundation stone of the Victoria Jubilee Wing of the Donnybrook Hospital for Incurables, on Wednesday, the 29th inst. His Excellency the Lord Lieutenant, and other distinguished persons, are taking great interest in the subject.

The Aberdeen Royal Infirmary Extension Fund is growing apace. Up to the present time the total amount intimated or paid is close upon £23,000. The Aberdonians are putting their shoulder to the wheel.

The Victoria Hospital at Bournemouth is to have a new building, the cost of which is not to exceed £7,000. The executive are about to advertise for tenders, and it is hoped the work will be put in hand forthwith.

A concert in aid of the Jubilee Extension Fund of the Dundee Royal Infirmary was given on Thursday evening at the Albert Hall. The programme included the cantata, *Queen Victoria and her Reign*, which was admirably rendered by a choir of about sixty boys, under the direction of Mr. Duncan Clarke. Lord Provost Henderson presided, and there was a large and appreciative audience.

The fourth annual meeting of the Paddington Green Children's Hospital was held on Friday, under the presidency of Mr. George Hanberry, chairman of the institution. The report read by the Rev. W. M. Carruthers stated that the subscriptions and donations had increased, and that a gratifying fact was the steady support of the working-classes. During the year the out-patients had contributed £133, whilst the larger parties of trade, temperance, and friendly societies had produced £104 12*s.* 10*d.*; £57 13*s.* 3*d.* had been received from the police of the F and X divisions as the result of a concert given by them. The existing accommodation for out-patients was very insufficient, and occasioned much overcrowding, and the committee had purchased the freehold at the rear of the hospital for £2,587, on which new rooms would be built for this special purpose; £2,000 was required, which it may be hoped will be shortly forthcoming.

Southampton has had its Hospital Sunday collections, and they do not show any indications of a Jubilee expansiveness. The total amount collected was £1,030 11*s.* 11*d.*, as against £1,060 10*s.* 0*d.* in 1886. The expenses were smaller than last year, and thus the balance was restored, there being a slightly larger sum for distribution in the present year. Still the story is not pleasing to the admirers of hospitals. Saving is no doubt good earning, but there is always a progressiveness about the needs of hospitals, and if the revenues be not also progressive—what then? The buildings are there, and the staff, but sorely needed work will be bound to remain undone. We cannot congratulate Southampton on its Jubilee Hospital collections.

The managers of the Worcester Infirmary have decided to enlarge their building, and to sell out stock to the amount of £3,500 for the purpose of defraying the cost. As a rule it is not pleasant to hear of funded property being disposed of for such a purpose, but the hospital is by no means poor, and after the sale of stock will still be endowed to the extent of over £50,000. The extension, which will add comparatively little to the cost of management, is very much needed, and will greatly augment the usefulness of the institution.

The Baroness Burdett-Coutts attended at an athletic festival at Tufnell Park on Thursday evening. The fête had been organised by the police and tradesmen of the Y Division for the benefit of the Building Fund of the Great Northern Central Hospital. In an interval of the sports Mr. Burdett-Coutts stood in his carriage and said the Baroness and himself were gratified to see how the tradesmen and working-classes were supporting the institution, which must be of immense benefit, directly and indirectly, to all. The Baroness was especially pleased to see the police working so energetically for the hospital. The cricket match, which was the first event of the day, was between the police and the tradesmen, and the result was 236 for the police, and only 36 for the tradesmen. It would be difficult to give a more striking evidence of the value of exercise as a health-factor than this simple cricket match between the police, who live and move in the open air, and the tradesmen who have their business in shops and factories.

The Governors of St. Mary's Hospital have decided to intermit the annual dinner this year in favour of a project which will doubtless command general approval and support. At present the hospital has no frontage in Praed Street, which is a great disadvantage. It is suggested that a few houses on the north side of Praed Street, adjoining the hospital, be purchased from the Grand Junction Canal Co., for the purpose of making an entrance there. If this be done, a large gateway can be made, through which passers-by could at least see the hospital, and in this way the institution would cease to be hidden in a corner. The enlargement would also give room for additional offices, nurses' residences, and special wards. A committee, with the Dean of the Medical School as chairman, has been formed for carrying out this very necessary object.

THE following vacancies are announced this week, the amount of the salary being placed in each case after the name of the institution :—

**Honorary Medical Officers.**

Manchester Hospital for Comsumption. Assistant Physician.
St. Pancras Dispensary. Physician.

**Resident Medical Officers.**

Birmingham General Hospital. Assistant House Surgeon. Qualified. No salary.
Durham County Hospital. House Surgeon. Qualified.—£100,
Hospital for Women, Soho Square. House Physician. Doubly-qualified.—£75.
Liverpool Dispensaries, 34, Moorfields. Assistant Surgeon. Registered.—£80.
Liverpool Southern Hospital. Ambulance Surgeon. One qualification or third-year Student.—No salary.
Prison Commissioners, 130, George Street, Edinburgh. Resident Surgeon for one of H.M. Prisons in Scotland.—£200.
St. Pancras Dispensary. One. Doubly-qualified.—£105.

**Non-resident Medical Officers.**

Midhurst Union. Two. Qualified.—£65 and £45.
Paddington Dispensary. One. Doubly-qualified.—£50.

**Matrons.**

Blackburn Infirmary. One.—£80.
Brentwood Cottage Hospital. One.—£25.
Burslem, Haywood Hospital. Matron and Visiting Nurse.
General Infirmary at Gloucester and the Gloucester Eye Institution. One.—£60.
Royal Portsmouth Hospital. One.—£80.

**Trained Nurses.**

Canterbury Nurses' Institute. Several.
Leeds Fever Hospital. Charge Nurse.—£30.
Richmond Hospital, Surrey. Night Nurse.—£20.
Truro Nurses' Home. One.

**Probationers.**

Radcliffe Infirmary, Oxford. Ladies.—Premium, £31 10*s*.

---

THE Secretary of the Dewsbury General Infirmary, Mr. C. J. Abbs, has developed the Hospital Saturday Fund in a new direction. Finding the convalescent fund £17 to the bad, he suggested that a few boxes should be placed in the streets on "Jubilee Day," and a special collection taken for it—this collection to be independent of the regular Saturday and Sunday collections. The Yorkshiremen thought the idea worthy of consideration, and are about to try it. Probably it will prove a happy inspiration, and if it should, perhaps other towns may be found willing to adopt and improve upon it.

THE Duke of Grafton presided at the 36th anniversary festival of the City Orthopædic Hospital, at the Holborn Restaurant. The institution, which is entirely free in principle, has treated 52,000 cases of deformity, most of which were in children. It appears the lease of the hospital is now expiring, and there is no prospect of its being renewed. The committee have set aside £2,000 as the nucleus of a fund for a new building. The secretary read a list of subscriptions and donations, amounting to £910, which he hoped would be increased to £1,000.

---

MR. JUSTICE KAY has issued an injunction to prevent the Middlesex Hospital erecting some buildings which would have interfered with an "ancient light" belonging to one Tooley. Although hospitals are institutions devoted to benevolence, it does not follow that the benevolence will always be shown to neighbours as well as to patients. Nevertheless, as they live by the goodwill of the public, they should always, if possible, so act as to increase rather than impair that sentiment in the public mind.

THE Marquis of Ripon, presiding last week at the annual dinner in aid of the Hospital for Sick Children, at Cromwell House, Highgate, said the recent history of the institution had been somewhat critical, and the managers found themselves with the prospect of a large deficit. An urgent appeal, however, had been made, and had met with such a generous response, that they might now regard their position, so far as annual income was concerned, as fairly satisfactory. A new wing was urgently needed, in order to afford separate wards for children suffering from diphtheria, whooping-cough, and other infectious diseases, as well as for accidents and very young infants. The Marquis stated that Mr. G. J. Goschen had formed a committee of ladies for the promotion of a Children's Jubilee Fund.

---

THE managers of the Torbay Hospital are either misrepresented by the local press, or need to go to school to learn the elements of politeness. Recently Mr. Sidney Hacker, the county coroner, held an inquiry at the hospital into the circumstances attending the death of Herbert Brown, aged five, who expired on the previous day from injury received by being run over. The inquiry, it seems, was held in the dentist's room, and the atmosphere was strongly impregnated with the smell of disinfectants. The coroner said he was sorry the jury were compelled to meet in such an unpleasant room, adding, the authorities apparently did not think it worth while to provide a decent place in which the inquest could be held. The only way in which they could mark their dissatisfaction was, that in the event of any of them voluntarily giving up their fees to a public institution, that institution should not be the Torbay Hospital. A juror asked if there was no board-room in the hospital. The coroner said that there was such a room, and that every other hospital in the district provided a room for inquests. We cannot congratulate the Torbay managers on their court manners.

---

THE Bishop of Bedford presided at the Sunday Fund meeting at the Shoreditch Town Hall on Saturday afternoon. He took the rather pessimistic view that hospitals might, in the near future, be compelled to haul down the voluntary flag and hand themselves over to the State, a possible catastrophe which he deeply deplored. If that occurred, "the sweet pleasure of voluntarily and charitably giving would be gone for ever, for no sentiment could attach to the compulsory payments of taxes or rates." There the Bishop hardly echoed the experience of many householders. In some cases, at least, a very decided sentiment attaches to the compulsory payment of taxes and rates. The sentiment is probably not so amiable as it might be, but that it exists we know of at least one person who can afford satisfactory evidence on quarter-day. No admirer of hospitals can wish them to take the same position in his thoughts as the police force or the vestry. We think, however, the Bishop took too gloomy a view of the matter. With the increase of knowledge we anticipate a widespread revival of practical interest in the whole subject of medical charities.

Hospital Saturday was favoured with Queen's weather, and a royal collection should have followed. The counting in all the districts is not yet completed as we go to press. If publicity be of service, the Saturday Fund managers have, like Willing, given the hospitals "bold advertisement." The metropolitan area was divided for the purposes of the collection into thirty districts, each with its own committee and band of willing workers. There were two thousand collecting stations, and the boxes distributed were over three thousand. Passengers by river and rail were asked to help the street and lane pedestrians. Cabmen, policemen, postmen, railway servants, members of workingmen's clubs, friendly and temperance societies, all entered heartily into the movement. A staff of fifty bank clerks, under the superintendence of Mr. W. H. Nicholls, manager of the Holborn branch of the City Bank, counted the gains of the central district at the City Temple. In other instances the counting was done by the local committees. The workshop collection is not yet completed. It is the largest and least fluctuating source of the Fund, and is expected to be greatly in excess of the amounts raised in former years. Forty thousand collecting sheets have been issued to more than twenty thousand business establishments. The workmen are rousing themselves with a will, to their own great credit, and to their advantage in every way.

The annual meeting of the committee and subscribers of the Central London Throat and Ear Hospital, treating also diseases of the nose, Gray's Inn Road, of which the Archbishop of Canterbury is the President, was held in the board-room of the hospital on Friday afternoon; Captain Hutton, chairman of the committee, presiding. The secretary, Mr. R. Kershaw, read the report, which showed that in the year under notice a larger amount of relief had been afforded than in any previous year. 5,434 new out-patients had been treated, against 4,946, and 315 in-patients had been admitted as against 237 in the preceding year. The benefit the hospital affords is not confined to its own locality, for the report showed that patients came from all parts of the kingdom, as well as from every metropolitan district. The income of the hospital had been £1,887 18*s*., which was a decrease of £310 14*s*. 8*d*. compared with last year's income. The provident system was still working with the most successful and encouraging results, the out-patients having contributed £773 16*s*., and the in-patients £196 12*s*. 6*d*. Larger grants had also been received from both the Hospital Sunday and Hospital Saturday Funds. The expenditure had been £1,946 17*s*. 1*d*., which exceeded the income by £56 19*s*. 1*d*. There was also a mortgage debt of £2,000, and in consequence of the death of one of the mortgagees, this liability had to be discharged not later than July. Each member of the committee and medical staff had given twenty-five guineas in response to the appeal which was made to defray this debt.

"A subject of great interest" (we quote from the annual report of the New England Hospital at Boston) "is the care of giving outdoor refreshment to our patients in summer, and even in winter. Any visitor to the hospital this summer must have seen with pleasure the number of patients sitting in easy-chairs or lying in cot-beds under the shelter of our little group of cedars, and drinking in the healing influences of sun and air. This treatment is not only very important for the recovery of our own patients, but also demonstrates the value of such natural hygienic influences; but, like all the best things of life, this good cannot be secured without much labour and expense. Many of the patients must be carried up and down stairs, and this makes us desire more than ever the convenience of an elevator, which would save much very hard work."

During the last fortnight an important series of meetings in aid of the Hospital Sunday Fund has been held in all the influential metropolitan and suburban centres. Speakers, representative of all classes of the people, from the Archbishop of Canterbury downwards, have attended. The meetings have not been large, but the speeches have, almost without exception, been intelligent, practical, and full of sympathy with the movement. It is clear either that the hospitals enjoy a very limited popularity, or that their more immediate friends lack the skill or the enthusiasm to evoke a hearty demonstration from the public. Sir Edmund Currie, at the Blackheath meeting, bewailed the poor attendance, and seemed sorry that he had not announced himself to speak with his face blackened, as in that case he felt sure his audience would have been overflowing. Somebody is to blame, and ought to be blamed severely. Is it the public, or is it the lukewarmness of hospital managers? Before another year, steps will have to be taken to bring about a very different state of matters.

A Cottage Hospital is in contemplation at Huntly, N.B. It appears to have been in contemplation rather a long time. The cautious Scots of the far North like to have the money in hand before giving orders for stone and lime. They should, however, be a little more plucky. A fellow-townsman of theirs in London has given largely to the building fund of one of the London hospitals. Perhaps he might be induced to "look at home," if he were asked very nicely. There are also some large landed proprietors in the neighbourhood of Huntly. What is the condition of their pockets? It ought not to be a difficult matter to raise four or five thousand pounds in the district for so excellent and necessary an object. Think over the matter again, men and women of Huntly!

It is rather a "far cry," as the Scotch have it, from Huntly to Bingley in Yorkshire. The Bingleyites are looking in the direction of a Cottage Hospital; and if we may judge from the names of the members of the committee, they will not look long before they begin to leap. Some of them we know to be men of substance, and others to be men of leisure. If the leisured ones will take up the matter very heartily, and give to it all their spare time, the hospital will soon be an accomplished fact. Bingley, though little known in the South, has a large manufacturing population, and must need a hospital very greatly. There is abundance of wealth in the neighbourhood; and the committee will do well to organise promptly, and "strike whilst the iron is hot."

